# Prevalence and related factors of symptom-based sexually transmitted infections among men who inject drugs: findings from three nation-wide surveys in Iran, 2010–2023

**DOI:** 10.1136/bmjopen-2025-110882

**Published:** 2026-09-22

**Authors:** Maliheh Sadat Bazrafshani, Soheil Mehmandoost, Fatemeh Tavakoli, Hossein Mirzaei, Naser Nasiri, Mahkameh Rafiee, Sakineh Narouee, Mehrdad Khezri, AliAkbar Haghdoost, Armita Shahesmaeili, Ali Mirzazadeh, Omid Zamani, Maryam Dashtizadeh, Zahra Gholamipour, Mohammad Karamouzian, Hamid Sharifi

**Affiliations:** 1 HIV/STI Surveillance Research Center, and WHO Collaborating Center for HIV Surveillance, Institute for Futures Studies in Health, Kerman University of Medical Sciences, Kerman, Iran (the Islamic Republic of); 2 Bio Environmental Health Research Center, Jiroft University of Medical Sciences, Jiroft, Iran (the Islamic Republic of); 3 Health Services Management Research Center, Institute for Futures Studies in Health, Kerman University of Medical Sciences, Kerman, Iran (the Islamic Republic of); 4 Department of Epidemiology, New York University School of Global Public Health, New York, New York, USA; 5 Social Determinants of Health Research Center, Institute for Futures Studies in Health, Kerman University of Medical Sciences, Kerman, Iran (the Islamic Republic of); 6 Department of Epidemiology and Biostatistics, University of California San Francisco, San Francisco, CA, USA; 7 World Health Organization, Tehran, Iran (the Islamic Republic of); 8 Vice-Chancellor for Health, Zahedan University of Medical Sciences, Zahedan, Iran (the Islamic Republic of); 9 Vice-Chancellor for Health, Iran University of Medical Sciences, Tehran, Iran (the Islamic Republic of); 10 Centre on Drug Policy Evaluation, MAP Centre for Urban Health Solutions, St Michael’s Hospital, Toronto, Ontario, Canada; 11 Dalla Lana School of Public Health, University of Toronto, Toronto, Ontario, Canada; 12 Knowledge Hub for Migrant and Refugee Health, Institute for Futures Studies in Health, Kerman University of Medical Sciences, Kerman, Iran (the Islamic Republic of)

**Keywords:** Sexually Transmitted Disease, Health Surveys, Public health

## Abstract

**Abstract:**

**Background:**

Sexually transmitted infections (STI) are a major public health concern and can increase HIV acquisition and transmission risk. People who inject drugs (PWID) may have elevated vulnerability due to overlapping sexual, injection-related and structural risk environments.

**Objective:**

To estimate the prevalence of symptom-based STI among PWID in 2010, 2014 and 2023 and explore factors associated with STI symptom reporting among men who inject drugs (MWID) in 2023.

**Design:**

Exploratory analysis of three repeated cross-sectional national bio-behavioural surveillance surveys.

**Setting:**

Major Iranian cities.

**Participants:**

PWID aged ≥18 years who had injected in the past 12 months, resided in the study city and provided informed consent. Facility/outreach recruitment was used in 2010/2014, and respondent-driven sampling (RDS) was used in 2023.

**Primary outcome:**

Self-reported pain/burning, genital ulcers, discharge or genital warts in the past 12 months defined symptom-based STI reporting. Data were collected by structured face-to-face interviews, followed by HIV testing.

**Results:**

Symptom-based STI prevalence among PWID was stable (2010: 10.1%; 2014: 10.5%; 2023: 10.3%). Among MWID in 2023, prevalence was 8.8% (95% CI 7.26 to 10.57). STI symptom reporting was associated with HIV and Hepatitis C Virus positivity. In exploratory multivariable analysis, STI symptom reporting was associated with being separated/widowed/divorced (AOR 2.56; 95% CI 1.14 to 5.7), multiple sexual partners (AOR 4.08; 95% CI 1.22 to 13.65), unprotected sex with casual partners (AOR 2.41; 95% CI 1.27 to 4.58), ever experienced sex with men (AOR 2.98; 95% CI 1.36 to 6.54), stimulant injection (AOR 2.57; 95% CI 1.08 to 6.14) and sharing injection equipment (AOR 2.84; 95% CI 1.53 to 5.27).

**Conclusion:**

Symptom-based STI remain persistent among PWID in Iran. Findings highlight the importance of supporting integrated STI prevention, testing, condom provision, syndromic management and linkage to care within harm reduction services to improve STI control among PWID. Associations should be interpreted as hypothesis-generating, not causal.

**STRENGTHS AND LIMITATIONS OF THIS STUDY**

- The study used data from three rounds of national bio-behavioural surveillance among people who inject drugs over a 13-year period, allowing assessment of temporal patterns in symptom-based sexually transmitted infections (STI) prevalence.
- Using structured and standardised questionnaires based on World Health Organization recommendations promotes consistency and comparability of data across sites and survey rounds.
- Symptom-based STI assessment may underestimate true prevalence due to the high proportion of asymptomatic infections, particularly among men.
- Differences in sampling methods across survey rounds may affect comparability of prevalence estimates over time.
- The cross-sectional design and exploratory modelling approach preclude causal inference.

## Background

 Sexually transmitted infections (STI) pose a significant global health challenge, affecting sexual health and reproductive morbidity and increasing the risk of HIV transmission.^1^ Although many STI are curable, they impose a substantial economic burden on healthcare systems.^2^ Globally, more than 1 million new STI cases occur each day among adults aged 15–49 years.^3^ Global statistics indicate an increasing trend in the number of STI cases between 1990 and 2019, with higher age-standardised incidence rates of trichomoniasis and genital herpes, particularly in Sub-Saharan Africa and East Asia.^4^ In Iran, STI symptom prevalence was notably high in 2019, affecting more than 80% of women and 33% of men during their sexually active lives.^5^

STI transmission primarily occurs through higher-risk sexual behaviours, including unprotected vaginal or anal sex and sex with partners diagnosed with STI. However, STI are particularly concerning among populations at increased risk of HIV, including men who have sex with men, sex workers^6^ and people who inject drugs (PWID).^7^ Indeed, the intersection of injection drug use and sexual risk behaviours creates a particularly complex epidemiological landscape. Among PWID, STI remain understudied despite this population’s elevated vulnerability shaped by sexual risk behaviours, injection-related risk environments and shared structural determinants.^8 9^ Recent evidence suggests a strong association between illicit drug use and STI incidence, particularly among young adults,^10^ with injection drug use associated with STI.^11^ PWID face dual risk pathways: blood-borne infections through contaminated injection equipment and STI through unsafe sexual practices, making them a critical population for surveillance and intervention.

Timely and accurate diagnosis is crucial for implementing effective targeted treatments and controlling disease spread within higher-risk groups,^12^ and STI treatment-seeking behaviours play an important role in STI control through appropriate diagnosis.^13^ However, in Iran, approximately one-third of men with STI symptoms take no action when experiencing common STI symptoms.^14^ Key populations, such as PWID, face multiple barriers to STI control, including limited public awareness, stigma, limited access to care, financial constraints and low STI risk perception.^15 16^ In response to these challenges, Iran has integrated syndromic STI screening with harm reduction services through drop-in centres (DICs) to enhance the diagnosis of STI among higher-risk groups. These screening services are available at DICs and harm reduction stations, where symptoms such as excessive discharge and genital ulcers are screened monthly among referred individuals.^17 18^ Iran’s plan includes integrating STI screening for key populations into general healthcare service centres to increase access to STI-related health services for these groups.^19^

Previous studies have identified sociodemographic characteristics, housing instability, marital status, income, sexual behaviours and injection-related practices as correlates of STI among PWID.^20 21^ Sex and gender may also shape both STI risk and care-seeking behaviours among PWID.^22^ Additionally, men who inject drugs (MWID) may face distinct barriers related to masculinity norms, stigma and reluctance to disclose sensitive sexual or drug-related behaviours, which may affect their health-seeking behaviours.^23^ However, evidence on symptom-based STI reporting and its associated factors among PWID, especially among Iranian men, remains limited. Given that men constitute the large majority of PWID in Iran and are more likely to access harm reduction services,^24^ understanding STI symptom reporting and associated factors among MWID is important for informing targeted interventions. In Iran, available studies provide only fragmented evidence, with limited examination of symptom-based STI among MWID. Therefore, the context-specific evidence base is insufficient to prespecify a minimally sufficient set of confounders for symptom-based STI reporting among MWID in Iran. We therefore considered an exploratory, hypothesis-generating approach appropriate. Accordingly, this study aimed to estimate trends in the prevalence of symptom-based STI among PWID from 2010 to 2023 and to explore factors associated with STI symptom reporting among MWID using data from the latest national survey of PWID in Iran.

## Methods

### Study overview

This study was an exploratory analysis of repeated cross-sectional national bio-behavioural surveillance surveys (BBSS) conducted among PWID in Iran. Participants were recruited from 10 major cities in 2010 and 2014 and 14 major cities in 2023. We used all three survey rounds to estimate trends in symptom-based STI prevalence. The 2023 survey was used for the exploratory analysis of factors associated with self-reported STI symptoms among sexually active MWID.

### Study participants and setting

The 2010 survey included 2546 PWID recruited from various facilities, including DICs, shelters, methadone maintenance treatment centres, voluntary counselling and testing centres, as well as outreach spots in 10 cities.^25^ In 2014, 2399 PWID were recruited in the second survey round. This survey covered the same 10 cities as the 2010 survey: Ahvaz, Tabriz, Kermanshah, Zahedan, Shiraz, Tehran, Kerman, Khorramabad, Mashhad and Sari.^26^ In these rounds, participants were recruited through a facility-based method. Eligibility criteria included being aged 18 years or older, having injected drugs in the past 12 months, residing in the study city and providing informed consent.

The most recent survey was conducted between May and August 2023 and recruited 2380 PWID from 14 major cities, including Tehran (central north), Karaj (central north), Tabriz (northwest), Sari (north), Mashhad (northeast), Yazd (central), Kermanshah (west), Khorramabad (west), Dorud (west), Ahvaz (southwest), Shiraz (south), Kerman (southeast), Saravan (southeast) and Zahedan (southeast). In this round, participants were recruited through a respondent-driven sampling (RDS) method following the same core eligibility criteria as the 2010 and 2014 rounds, with the additional RDS-specific requirement that non-seed participants present a valid recruitment coupon. RDS is a network-based recruitment method commonly used to reach hidden or hard-to-reach populations, including PWID.^27^ In this method, referral coupons are used to recruit participants. Well-networked participants were selected as ‘seeds’, and initial recruitment was performed through them.^28^ Seeds were trained to recruit their peers for the survey, and recruitment continued until the required sample size was achieved. Participants received three coupons with a 3-week validity period. Each coupon had a unique number, interview site address, contact information, interview site’s operational hours and an expiry date. If a referred person was eligible for the study and presented a coupon within 3 weeks, they were enrolled as a participant. Further details of this approach have been previously published.^29^

### Data collection

In all three survey rounds of BBSS, data were collected using structured, standardised questionnaires based on WHO recommendations.^30^ The questionnaire used in the 2023 round was updated according to WHO bio-behavioural survey guidance published in 2021.^31^ Details of previous rounds were published elsewhere.^25 32 33^ Face-to-face interviews were conducted by trained gender-matched interviewers. Before participation, verbal informed consent was obtained from all participants. To ensure privacy, confidentiality and a comfortable environment, interviews were held in a private room. All information, including sociodemographic data and details on sexual practices, drug injection and health services, was gathered through these interviews. Following pretest counselling and consent for testing, participants completed the questionnaire and then underwent HIV testing. Two different brands of rapid HIV tests were used: SD-Bioline rapid tests from South Korea, with reactive results confirmed by a follow-up Unigold HIV rapid test. Participants who declined HIV testing could still complete the behavioural questionnaire.

### Outcome

The outcome was self-reported, symptom-based STI in the past 12 months. Participants were asked whether they had experienced any STI-related symptoms, including pain or burning, genital ulcers, excessive discharge or genital warts. Participants could report more than one symptom. Those reporting at least one symptom were classified as reporting symptom-based STI. This outcome reflects self-reported symptoms and should not be interpreted as laboratory-confirmed STI infection. Participants with missing responses to this question were excluded from the analysis.

### Covariates

#### Conceptual framework

Candidate covariates were organised using the Risk Environment Framework, which conceptualises drug-related harms as arising from interactions between individual behaviours and broader physical, social, economic and policy environments. This framework was used to ensure that the exploratory analysis considered sociodemographic, service-related, sexual, injection-related and structural factors relevant to PWID.^34–36^

Sociodemographic variables included age, categorised as <30 years and ≥30 years; education, categorised as less than high school versus high school or higher education; marital status, categorised as single, married or living with a sexual partner/temporary marriage or separated/widowed/divorced and monthly income, categorised as <US$70 versus ≥US$70. Structural and social vulnerability variables included homelessness in the last 12 months, incarceration in the last 12 months, and having current health insurance. Service-related variables included ever tested for HIV, ever tested for Hepatitis C Virus (HCV) and receipt of harm reduction services in the last 12 months.

Sexual risk behaviours included having multiple sexual partners in the last 6 months, unprotected sex with a casual partner in the last 6 months and ever having had sex with men. Injection-related variables included the type of main drug injected in the last 6 months, categorised as opioids or stimulants, daily injection in the last 6 months and sharing needles, syringes or other injection equipment in the last 6 months. Injection-related variables were included as markers of broader risk environments, social network characteristics and potentially unmeasured sexual risk behaviours, rather than as presumed direct transmission routes for STI.^34–36^

### Data analysis

The analytic sample for the regression analysis was restricted to MWID in the 2023 survey who reported sexual contact with any partner in the past 6 months. Continuous variables were summarised using means and SDs, and categorical variables were summarised using frequencies and percentages. Prevalence estimates were reported with 95% CIs. Missing values were not imputed; analyses were conducted using available data for each variable, and participants with missing STI outcome data were excluded from outcome-specific analyses. Data from the 2010, 2014 and 2023 surveys were used to describe trends in symptom-based STI prevalence. Data from the 2023 survey were used for the exploratory analysis of factors associated with STI symptom reporting among sexually active MWID. Because evidence on determinants of symptom-based STI reporting among MWID in Iran is limited, we used an exploratory variable-selection approach. First, bivariable logistic regression was used to examine crude associations between candidate covariates and STI symptom reporting. Variables associated with STI symptom reporting at p<0.20 in bivariable analyses were entered into a multivariable logistic regression model.^37^ Backward stepwise selection was then used to obtain a parsimonious model of adjusted associations, with likelihood ratio tests used to compare reduced and full models at each step. This approach was used for hypothesis generation and should not be interpreted as identifying a minimally sufficient adjustment set or estimating causal effects.

Collinearity among variables entered into the multivariable model was assessed using variance inflation factors (VIFs) and tolerance statistics.^38^ VIF values ranged from 1.04 to 1.25, with a median VIF of 1.12, suggesting no substantial statistical collinearity.^38^ We also considered conceptual overlap among variables, particularly among sexual risk behaviours, injection-related behaviours and service-use indicators. Conceptually related variables were retained when they represented distinct dimensions of the risk environment.

Adjusted ORs (AORs) and 95% CIs were reported for variables retained in the final exploratory model. These estimates are interpreted as adjusted associations only and not as causal effects. χ^2^ tests were used to examine associations between HIV, HCV and Hepatitis B Virus (HBV) status and STI symptom reporting. Statistical significance was set at a p value of 0.05 or less. All analyses were conducted using Stata V.17.

### Patients and public involvement

Patients and members of the public were not involved in the design, conduct, reporting or dissemination of this research.

## Results

### Overall characteristics of PWID

In the present study, 7325 PWID were recruited across three survey rounds: 2546 in 2010, 2399 in 2014 and 2380 in 2023. The majority were men (7094, 96.8%). The mean age was 37.9 years (SD 9.6), and 5524 (75.4%) had a high school education or less.

### Overall trends in symptom-based STI prevalence

Across survey rounds, the number of PWID who reported sexual contact in the last 6 months was 1124 (44.1%) in 2010, 1124 (46.8%) in 2014 and 1257 (52.8%) in 2023. Across these rounds, the prevalence of symptom-based STI remained relatively stable (2010: 10.1% (95% CI 8.35 to 11.96); 2014: 10.5% (95% CI 8.76 to 12.43) and 2023: 10.3% (95% CI 8.63 to 12.07)). The prevalence of genital ulcers and excessive discharge among symptomatic PWID was 64.6% and 76.1%, respectively, in 2010. In 2014, genital ulcers remained the most commonly reported symptom among symptomatic PWID (94.9%). In 2023, pain and burning were the most commonly reported symptoms among symptomatic PWID (90.7%), whereas genital ulcers and genital warts had lower prevalence in this round (11.6% and 0.8%, respectively) (table 1).

**Table 1:** Prevalence and treatment of sexually transmitted infection symptoms in three rounds of BBSS among PWIDs in Iran

Year	2010		2014		2023	
Variable	Total (2546)	Men (2480)	Total (2399)	Men (2333)	Total (2380)	Men (2281)
Participants with a recent history of sexual contact in the last 6 months	1124 (44.1)	1086 (43.8)	1124 (46.8)	1109 (47.5)	1257 (52.8)	1191 (52.2)
Participants with recent STI symptoms in the last 12 months (symptomatic PWID)	113 (4.4)	94 (3.8)	118 (4.9)	117 (5.0)	129 (5.4)	105 (4.6)
Prevalence of STI among sexually active PWID (95% CI*)	10.1 (8.35–11.96)	8.7 (7.05–10.48)	10.5 (8.76–12.43)	10.6 (8.80–12.50)	10.3 (8.63–12.07)	8.8 (7.26–10.57)
Common symptoms among symptomatic PWID, n (%) (multiple choice)†						
Pain and burning	NR‡	NR	NR	NR	117 (90.7)	98 (93.3)
Genital ulcers	73 (64.6)	58 (61.7)	112 (94.9)	111 (94.9)	15 (11.6)	12 (11.4)
Excessive discharge	86 (76.1)	69 (73.4)	6 (5.1)	6 (5.1)	23 (17.8)	10 (9.5)
Genital warts	NR	NR	NR	NR	1 (0.8)	1 (1.0)
Seeking treatment among symptomatic PWID, n (%)§						
Yes	56 (49.5)	48 (51.1)	70 (59.3)	70 (59.8)	96 (74.4)	77 (73.3)
No	56 (49.5)	45 (47.9)	46 (39.0)	46 (39.3)	31 (24.0)	26 (24.8)
Type of treatment, n (%) (multiple choice)¶						
Referred to a pharmacy/drugstore and received pharmacist counselling	2 (3.6)	2 (4.2)	13 (18.6)	13 (18.6)	15 (15.6)	9 (8.6)
Sought advice from friends, used herbal medicine or self-medicated	13 (23.2)	12 (25.0)	12 (17.1)	12 (17.1)	70 (72.9)	57 (54.3)
Referred to governmental or private medical clinics and harm reduction centres	40 (71.4)	32 (66.7)	47 (67.1)	47 (67.1)	28 (29.2)	22 (20.9)

* Prevalence was based on two symptoms (genital ulcers and excessive discharge).

† Percentages for specific STI symptoms (genital ulcers, excessive discharge, pain/burning and genital warts) are calculated among symptomatic PWID who reported any STI symptom.

‡ Not reported (NR).

§ Treatment-seeking percentages are also calculated among symptomatic PWID.

¶ Denominator for the type of treatment method was those who sought treatment.

BBSS, bio-behavioural surveillance surveys; PWID, people who inject drugs; STI, sexually transmitted infections.

Across the three rounds, 49.5%, 59.3% and 74.4% of symptomatic PWID sought treatment for STI symptoms. In 2010 and 2014, seeking care from governmental or private medical clinics and harm reduction centres was the most frequently reported (71.4% and 67.1%, respectively). In 2023, seeking advice from friends, using herbal medicine, or self-medicating was frequently reported (72.9%) among symptomatic PWID (table 1).

### Overall symptom-based STI cascade of care in 2023

**Figure 1:**
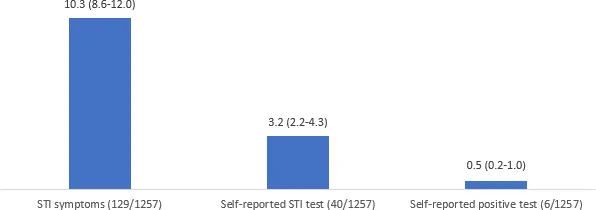
The sexually transmitted infection cascade of care among sexually active PWID in Iran, 2023. STI, sexually transmitted infections; PWID, people who inject drugs.

Of 1257 sexually active PWID, 129 (10.3%) had reported STI symptoms, 40 (3.2%) had previously been tested for STI, and 6 (0.5%) had reported testing positive for an STI (figure 1). Of those diagnosed, 5 (83.3%) had received treatment, and only 1 (20.0%) reported recovery.

### Characteristics of MWID in 2023

Among 1191 sexually active MWID surveyed, 95.6% were aged 30 years or older. Over a third (33.0%) were married, and 65.7% had less than a high school education. Homelessness and incarceration in the last 12 months were reported by 45.8% and 9.2%, respectively. Among MWID, 3.7% had a male sexual partner at last sex; 10.7% had ever had sex with men, and 43.3% had reported unprotected sex at last sex with a casual partner in the last 6 months. More than half received harm reduction services in the last 12 months (71.5%). Most of them injected opioids (81.4%); less than half (45.8%) reported daily injection, and 25.7% reported sharing needles, syringes or other injection equipment in the last 6 months (table 2).

**Table 2:** Prevalence of self-reported symptom-based sexually transmitted infections according to characteristics of MWID in Iran, 2023 (N=1191)

Variables	Level of variables	Men n=1191 (100%)	Prevalence of STI symptoms among men (95% CIs)	Crude OR (95% CI)	P value
Age groups	<30	53 (4.4)	7.5 (2.10 to 18.21)	Ref	–
	≥30	1137 (95.6)	8.9 (7.69 to 11.40)	1.19 (0.42 to 3.37)	0.738
Marital status	Single	278 (23.3)	4.0 (1.99 to 6.96)	Ref	–
	Married	393 (33.0)	6.6 (4.36 to 9.54)	1.71 (0.83 to 3.54)	0.141
	Separated, widowed or divorced	489 (41.1)	12.9 (10.04 to 16.18)	3.58 (1.85 to 6.93)	<0.001
	Living with a sexual partner or temporary marriage	31 (2.6)	16.1 (5.45 to 33.72)	4.66 (1.50 to 14.46)	<0.001
Educational level	Less than high school	782 (65.7)	10.7 (8.65 to 13.12)	2.22 (1.35 to 3.64)	0.002
	High school or higher education	409 (34.3)	5.1 (3.20 to 7.74)	Ref	–
Monthly income	<US$70	702 (59.3)	10.0 (7.85 to 12.43)	Ref	–
	≥US$70	482 (40.7)	7.3 (5.10 to 9.95)	0.70 (0.42 to 1.07)	0.108
History of incarceration in the last 12 months	No	1081 (90.8)	8.8 (7.16 to 10.63)	Ref	–
	Yes	110 (9.2)	9.1 (4.44 to 16.08)	1.03 (0.52 to 2.05)	0.915
History of homelessness in the last 12 months	No	646 (54.2)	5.6 (3.93 to 7.63)	Ref	–
	Yes	545 (45.8)	12.7 (9.98 to 15.74)	2.46 (1.61 to 3.73)	<0.001
Received harm reduction services in the last 12 months	No	339 (28.5)	7.7 (5.07 to 11.03)	Ref	–
	Yes	851 (71.5)	9.3 (7.41 to 11.43)	1.23 (0.77 to 1.95)	0.376
Ever tested for HIV	No	218 (18.3)	6.4 (3.55 to 10.54)	Ref	–
	Yes	961 (80.7)	9.4 (7.59 to 11.38)	1.50 (0.84 to 2.69)	0.169
Ever tested for HCV	No	865 (72.6)	10.3 (8.34 to 12.50)	Ref	–
	Yes	303 (25.4)	5.3 (3.04 to 8.43)	0.48 (0.28 to 0.84)	0.010
Current health insurance	No	915 (76.8)	9.3 (7.48 to 11.35)	Ref	–
	Yes	269 (22.6)	7.4 (4.60 to 11.24)	0.78 (0.47 to 1.30)	0.348
Multiple sexual partnerships in the last 6 months	No	634 (53.2)	5.8 (4.14 to 7.95)	Ref	–
	Yes	557 (46.8)	12.2 (9.60 to 15.21)	2.24 (1.47 to 3.40)	<0.001
Ever had sex with men	No	1055 (88.6)	7.9 (6.31 to 9.65)	Ref	–
	Yes	128 (10.7)	15.6 (9.81 to 23.09)	2.16 (1.28 to 3.67)	0.004
Unprotected sex at last sex with a casual partner in the last 6 months	No	366 (56.7)	4.6 (2.72 to 7.33)	Ref	–
	Yes	280 (43.3)	17.1 (12.91 to 22.07)	4.27 (2.38 to 7.56)	<0.001
Type of main injected drug in the last 6 months	Opioids	969 (81.4)	8.5 (6.78 to 10.39)	Ref	–
	Stimulants	54 (4.5)	24.1 (13.48 to 37.64)	3.42 (1.76 to 6.65)	<0.001
Daily injection in the last 6 months	No	443 (37.2)	6.8 (4.61 to 9.52)	Ref	–
	Yes	545 (45.8)	12.3 (9.65 to 15.34)	1.92 (1.23 to 3.02)	0.004
Sharing needles, syringes or other injection equipment	No	882 (74.1)	6.5 (4.93 to 8.29)	Ref	–
	Yes	306 (25.7)	15.7 (11.79 to 20.25)	2.69 (1.78 to 4.05)	<0.001

MWID, men who inject drugs; STI, sexually transmitted infections.

### Prevalence of symptom-based STI among MWID in 2023

The prevalence of symptom-based STI among MWID was 8.8% (95% CI 7.26 to 10.57). In bivariable comparisons, symptom-based STI prevalence differed across several sociodemographic, sexual, service-related and injection-related characteristics. Prevalence was higher among MWID who were separated, widowed or divorced (p value <0.001), lived with a sexual partner or were in a temporary marriage (p value <0.001), had less than high school education (p value=0.002) and reported homelessness in the last 12 months (p value <0.001). Symptom-based STI was also higher among MWID who reported multiple sexual partnerships in the last 6 months (p value <0.001), unprotected sex at last sex with a casual partner in the last 6 months (p value <0.001) and ever having had sex with men (p value=0.004). Higher prevalence was also observed among those who reported stimulant injection as the main injected drug in the last 6 months (p value <0.001), reported daily injection in the last 6 months (p value=0.004) and reported sharing needles, syringes or other injection equipment in the last 6 months (p value<0.001).

### Exploratory analysis of factors associated with symptom-based STI among MWID in 2023

In the final exploratory multivariable logistic regression model, several variables were associated with higher odds of reporting symptom-based STI among MWID: being separated, widowed or divorced (AOR 2.56; 95% CI 1.14 to 5.76) versus being single; multiple sexual partnerships in the last 6 months (AOR 4.08; 95% CI 1.22 to 13.65) versus having a single partner; unprotected sex at last sex with a casual partner in the last sex in the last 6 months (AOR 2.41; 95% CI 1.27 to 4.58) versus protected sex with casual partners at last sex and having ever experienced sex with men (AOR 2.98; 95% CI 1.36 to 6.54) versus never having had sex with men.

Injection-related variables were also retained in the final exploratory model and were associated with higher odds of reporting symptom-based STI among MWID: stimulant injection as the main injected drug in the last 6 months (AOR 2.57; 95% CI 1.08 to 6.14) versus opioid injection and sharing needles, syringes or other injection equipment in the last 6 months (AOR 2.84; 95% CI 1.53 to 5.27) versus not sharing injection equipment. These estimates should be interpreted as adjusted associations from an exploratory model rather than as causal effects or definitive predictors. Educational level, monthly income, homelessness in the last 12 months, ever having been tested for HIV, ever having been tested for HCV and daily injection in the last 6 months were not retained in the final exploratory model (table 3).

**Table 3:** Exploratory multivariable logistic regression model of symptom-based sexually transmitted infections among MWID in Iran, 2023

Variables	Levels of variables	Adjusted OR (95% CI)	P value
Marital status	Single	Ref	–
	Married	0.68 (0.19 to 2.47)	0.565
	Separated, widowed or divorced	2.56 (1.14 to 5.76)	0.023
	Living with a sexual partner or temporary marriage	1.30 (0.22 to 7.46)	0.766
Multiple sexual partnership in the last 6 months	No	Ref	–
	Yes	4.08 (1.22 to 13.65)	0.022
Unprotected sex at last sex with a casual partner in the last 6 months	No	Ref	–
	Yes	2.41 (1.27 to 4.58)	0.007
Ever experienced sex with men	No	Ref	–
	Yes	2.98 (1.36 to 6.54)	0.006
Type of main injected drug in the last 6 months	Opioids	Ref	–
	Stimulants	2.57 (1.08 to 6.14)	0.033
Sharing needles, syringes or other injection equipment in the last 6 months	No	Ref	–
	Yes	2.84 (1.53 to 5.27)	0.001

A total of **574 observations** were included in the final regression model. The analysis was restricted to participants with complete data for all variables included in the model; no imputation of missing data was performed.

MWID, men who inject drugs.

### Associations between symptom-based STI reporting and HIV, HBV and HCV status among MWID in 2023

STI symptom reporting was associated with HCV positivity and HIV positivity: STI symptoms occurred in 12.2% of HCV-positive vs 4.3% of HCV-negative participants (p value=0.038), and 27.3% of HIV-positive vs 8.6% of HIV-negative participants (p value=0.030). No statistically significant association was observed between HBV status and STI symptom reporting (table 4).

**Table 4:** Association between HBV, HCV and HIV status and symptom-based STI reporting among MWID in Iran, 2023

Variables	Levels of variables	Symptom-based STI		χ^2^ value	P value
		Negative	Positive		
HBV status	Negative (n=249)	231 (92.8)	18 (7.2)	–	0.324*
	Positive (n=5)	4 (80.0)	1 (20.0)		
HCV status	Negative (n=256)	245 (95.7)	11 (4.3)	4.32	0.038
	Positive (n=41)	36 (87.8)	5 (12.2)		
HIV status	Negative (n=1180)	1078 (91.4)	102 (8.6)	4.70	0.030
	Positive (n=11)	8 (72.7)	3 (27.3)		

*P-value derived from Fisher’s Exact Test.

STI, sexually transmitted infections.

## Discussion

This study provides important insights into the prevalence of STI symptoms among PWID in Iran, suggesting both persistent symptom burden and evolving patterns in care-seeking behaviours. Self-reported STI symptoms remain common among PWID, with approximately one out of 10 PWID reporting STI symptoms consistently over time. More than half of symptomatic PWID sought treatment for STI symptoms, though treatment approaches varied considerably across survey periods. In the exploratory multivariable analysis among sexually active MWID in 2023, STI symptom reporting was associated with several sexual, sociodemographic and injection-related characteristics, including multiple sexual partners, unprotected sex with casual partners, having ever had sex with men, being separated, widowed or divorced, stimulant injection and sharing needles, syringes or other injection equipment. Additionally, STI symptom reporting was more common among MWID with HIV or HCV positivity, highlighting the importance of integrated care approaches. Given the exploratory modelling strategy and cross-sectional design, these associations should be interpreted as hypothesis-generating rather than causal effects or definitive predictors.

The prevalence of symptom-based STI among PWID across the 13-year period was consistently around 10%. This stable trend suggests that STI prevention, diagnosis and treatment needs have persisted in this marginalised population. However, differences in sampling strategies across survey rounds should be considered when interpreting temporal comparisons. Limited comparable information exists on STI prevalence based on symptom reporting. Among the general population in Iran, 2.8% of men reported at least one STI symptom in 2014,^39^ indicating a substantially higher symptom burden among male PWID, although differences in population, measurement and survey methods limit direct comparison. Internationally, prevalence estimates vary substantially by region and methodology. In India between 2000 and 2007, 1% of PWID reported a history of either urethral discharge or genital ulcers as symptoms of STI.^40 41^ The prevalence of self-reported STI symptoms in Africa varied between 17% and 22% among PWID during 2011–2018.^42 43^ Our estimate was higher than some reports from India but lower than estimates reported in some African settings, underscoring the importance of differences in geography, study period, symptom definitions and sampling methods. Expanding harm reduction services, such as rapid STI testing where feasible, syndromic management in resource-limited settings and effective referral and linkage to treatment, may improve STI control among PWID.

A notable finding of our study was the change in care-seeking behaviours and treatment preferences among PWID with STI symptoms. More than half of PWID who reported STI symptoms sought treatment. In 2023, this proportion reached ~75%, with a notable shift towards seeking advice from friends, using herbal medicine and self-medicating. While increased care-seeking is encouraging, the shift away from formal healthcare settings raises concerns about treatment appropriateness and the potential for inappropriate antimicrobial use. In Iran, symptom-based STI screening has been integrated into harm reduction services,^44^ and PWID with STI symptoms are intended to be referred to governmental medical clinics. This trend may reflect changes in awareness of STI symptoms, distrust of formal healthcare systems,^45^ increased stigma experiences^46^ or perceived barriers to accessing quality care.^47^ Although STI treatment services are free, raising awareness of available services and referral pathways may help improve timely diagnosis, appropriate treatment and linkage to care.

We found that having multiple sexual partners in the last 6 months was associated with higher odds of reporting STI symptoms in the exploratory multivariable model, consistent with an established body of epidemiological evidence.^48 49^ These individuals may have more opportunities for exposure through sexual networks, particularly when condomless sex or partnerships with higher-risk partners occur.^50 51^ Providing sexual health information, accessible testing and treatment and preventive and supportive care may improve STI prevention and care.^52^ Conducting regular training sessions on safer sex and distributing educational brochures at DICs may improve the knowledge of individuals served by these centres. However, our findings extend beyond individual behavioural factors. The association with being separated, widowed or divorced and ever having had sex with men may reflect overlapping social vulnerabilities, sexual network characteristics, stigma and barriers to prevention or care among some MWID. Indeed, in addition to focusing on individual-level interventions, addressing structural barriers—such as ensuring consistent condom availability and reducing healthcare stigma—is essential for creating environments where PWID can access comprehensive sexual healthcare, regardless of their drug use status.

Injection-related characteristics were also retained in the final exploratory model. In particular, sharing needles, syringes or other injection equipment was associated with STI symptom reporting even after adjustment for measured sexual behaviours included in the model. This association should not be interpreted as evidence that sharing injection equipment directly transmits STI, such as gonorrhoea, chlamydia or syphilis, which are primarily transmitted through sexual contact. Rather, equipment sharing may be a marker of broader risk environments, including higher-risk social or drug-use networks, unstable access to harm reduction resources, overlapping sexual and injection partnerships or unmeasured sexual behaviours not captured in the survey. This interpretation is consistent with evidence that unsafe injection and sexual risk behaviours can co-occur among PWID.^26^ These findings suggest that STI prevention for MWID should not focus only on individual sexual behaviours but should also address the broader risk environment through integrated harm reduction, condom provision, STI testing or syndromic management, counselling and linkage to care.

Our findings show that STI symptom reporting was more common among MWID with HIV or HCV positivity. Because STI status in this study was symptom-based rather than laboratory-confirmed, these findings should not be interpreted as confirmed STI/HIV or STI/HCV coinfection prevalence. Instead, they suggest overlapping vulnerability and support the need for integrated clinical approaches. These findings align with previous research suggesting that sexual transmission contributes to at least 10% of new HIV cases among PWID, underscoring the importance of addressing sexual health within HIV prevention and care for PWID.^22^ Previous evidence has also reported a high prevalence of STI around the time of HIV diagnosis, supporting the role of STI prevention and treatment within HIV prevention strategies.^53^ The biological mechanisms underlying this association include STI-induced genital inflammation and immune activation, which may increase HIV susceptibility and transmission.^22^ More than a quarter of participants with positive HIV status had symptoms of STI, a proportion higher than STI/HIV co-occurrence estimates reported in Iran’s general population (2.6%–9.7%), although comparisons should be made cautiously because our STI measure was symptom-based.^54 55^ Integrating STI services with HIV and HCV care may improve the identification, management and treatment of overlapping infection risks within healthcare systems.^56^ This approach helps ensure that patients receive holistic and timely medical attention. Such a strategy may address both immediate treatment needs and the broader implications of overlapping infection risk and symptom burden, while improving linkage to prevention, testing and care for STI, HIV and HCV.

This study has several limitations that should be considered when interpreting the results. First, our reliance on symptom-based STI assessment may underestimate true prevalence, as many STI are asymptomatic, particularly in men. Second, reporting and recall bias may have also led to an underestimation of symptom-based STI prevalence among PWID. Third, the different sampling methodologies across survey rounds (ie, facility-based in 2010/2014 vs RDS in 2023) may affect the comparability of prevalence estimates. Moreover, our reliance on facility-based sampling in earlier rounds and RDS in 2023, while appropriate for hard-to-reach populations, may limit generalisability to PWID who remain completely hidden from health services. Fourth, this cross-sectional study was unable to establish causal relationships between the independent variables and a history of symptom-based STI due to the potential for reverse causation. Fifth, in this study, STI symptoms were assessed over the past 12 months, whereas sexual activity was assessed over the last 6 months. This approach may have led to the exclusion of PWID who experienced symptoms but were not sexually active during the last 6 months. Sixth, although candidate variables were informed by the Risk Environment Framework, using bivariable screening and stepwise regression methods may have led to the exclusion of important potential confounders that did not meet statistical significance thresholds, potentially resulting in residual confounding. Therefore, using an exploratory approach led to a model that was primarily data-driven rather than theory-informed, which may limit the robustness and causal interpretability of the identified associations. As a result, the findings should be interpreted with caution, and the observed relationships should be considered hypothesis-generating rather than confirmatory. Lastly, social desirability bias, stemming from face-to-face interviews, particularly concerning sensitive questions about drug use and sexual behaviour, could affect the accuracy of self-reported information. To mitigate some of these limitations, all interviews were conducted by gender-matched, trained interviewers in a private setting.

## Conclusion

This study highlights that symptom-based STI represent a persistent public health challenge among PWID in Iran. The relatively stable prevalence over 13 years suggests a need for enhanced prevention strategies. The data also point to concerning trends, including increased reliance on informal treatment and higher HIV and HCV positivity among MWID reporting STI symptoms. These findings underscore the critical need for integrated sexual health services within harm reduction programmes, improved healthcare accessibility and comprehensive approaches that address both sexual risk behaviours and broader risk environments among PWID. Due to the asymptomatic nature of many STI, increasing access to services such as rapid STI testing, free condoms and evidence-based educational interventions about safer sexual behaviours is warranted to improve STI control among PWID. Given the exploratory nature of the modelling approach, the associated factors observed in this study should be used to inform future research and intervention planning rather than interpreted as definitive causal determinants of STI risk. Future research should prioritise etiological approaches alongside syndromic surveillance to better understand the true burden of STI in this population and to evaluate integrated intervention strategies.

## Data Availability

Data are owned by the Ministry of Health and Medical Education. Data may be made available upon reasonable request to the senior author (Hamid Sharifi;hsharifi@kmu.ac.ir) for researchers who meet the criteria for access to confidential data set by Iran’s Ministry of Health and Medical Education.
