# Supplementary material for: Prevalence and related factors of symptom-based sexually transmitted infections among men who inject drugs: findings from three nation-wide surveys in Iran, 2010–2023
Source: BMJ Open. 2026 Sep 22;16(9):e110882. doi: 10.1136/bmjopen-2025-110882 (PMC13599987; doi:10.1136/bmjopen-2025-110882)
Supplement: Supplementary file 1 [file bmjopen-2025-110882.reviewer_comments.pdf]

PEER REVIEW HISTORY

BMJ Open publishes all reviews undertaken for accepted manuscripts. Reviewers are asked to complete a checklist review form and are provided with free text boxes to elaborate on their assessment. These free text comments are reproduced below.

ARTICLE DETAILS

Title (Provisional)

Prevalence and related factors of symptom-based sexually transmitted infections among men who inject drugs: Findings from three nationwide surveys in Iran, 2010-2023

Authors

Bazrafshan, Maliheh Sadat; Mehmandoost, Soheil; Tavakoli, Fatemeh; Mirzaei, Hossein; Nasiri, Naser; Rafiee, Mahkameh; Narouee, Sakineh; Khezri, Mehrdad; Haghdoost, AliAkbar; Shahesmaeili, Armita; Mirzazadeh, Ali; Zamani, Omid; Dashtizadeh, Maryam; Gholamipour, Zahra; Karamouzian, Mohammad; Sharifi, Hamid

---

VERSION 1 - REVIEW

**Reviewer**	**1**
**Name**	**Dack, Kyle**
**Affiliation**	**University of Bristol, MRC Integrative Epidemiology Unit**
**Date**	**22-Oct-2025**
**COI**	**None**

Statistical review summary:

Overall this is a very well written paper. The methods are described clearly and the interpretation of the findings is balanced. However, the modelling building practice has a high risk of bias because it is data-driven without theoretical justification, and risks introducing confounding bias and inflated type 1 error. I recommend replacing this with an evidence-based approach to variable selection, such as discussed for example here: https://pmc.ncbi.nlm.nih.gov/articles/PMC6447501/ , https://pubmed.ncbi.nlm.nih.gov/37771042/

1. Study framing: The authors acknowledge in the discussion that the data type prevents causal interpretation because all measurements were taken at the same time. For the most part, the interpretation and reporting is aligned with this. However, the word “influence” is used in various parts of the manuscript which does imply causality. I suggest alternatives such as “correlate”, “explain variance in”, “linked to”, etc.

Abstract:

2. In the results where it states “Also in this year” it is not clear what year is being referred to.

Methods:

3. I suggest adding a “study overview” or “study design” section to begin the methods, giving brief details of the design (repeated cross-sectional?), primary outcome, study dates, and participants. This is described in more detail in the STROBE elaboration document, and helps readers quickly understand the basic elements of the study.

4. Overall the methods are clear, reasonable, and transparent. My main concern is the variable selection strategy. Section 2.4 lists numerous variables, but there is not a distinction between independent variables theorized to correlate with the outcome, and covariates included to minimize confounding bias.

The current approach screens all variables by bivariate significance, and includes the significant variables in the final multivariable model. This is essentially a hypothesis-free approach, it is data-driven and does not account for confounding bias. It also introduces the risk of other forms of bias such as overfitting and inflated type 1 error. Variable selection should instead by guided by prior evidence and theory, with covariates selected to minimize confounding bias. If prior evidence/theory is not available, I recommend explicitly reframing the analysis as exploratory and descriptive, due to the higher risk of bias.

5. “A multivariable logistic regression model was performed to report STI symptoms and adjusted odds ratios (AORs).” I suggest specifying the full model parameterization – i.e. which variables were included.

6. It is not clear how time is handled in the analyses. It looks to me like the chi-square bivariate comparisons, and the regression models were all limited to 2023 data. I recommend stating this for clarity, and to reassure readers because my initial concern was that the models may pool data from the 3 surveys, which would break the assumption of non-clustered data.

I suggest also clarifying what the 2010 and 2013 data was used for, i.e. the trend analyses shown in section 3.1.

Results

7. Please begin by repeating the sample sizes, and the basic characteristics of the cohorts, to align with STROBE requirements.

8. Section 3.5. I suggest adding more information in this section. Currently it does not add much which cannot already be found in the corresponding table, and again I do not think it fully adheres to STROBE in relation to comprehensive explanation of the statistics. E.g. “Had multiple sexual partnership in the last 6 months (AOR: 4.42; 95% CI: 1.31 – 14.88);” – explain the comparison category, explain the meaning of a 4.42 odds ratio, acknowledge the very

wide confidence interval which suggests uncertainty in the precise point estimate, but indicates evidence of some form of increased odds. There is also no comment on the variables which were not significant, which risks introducing reporting bias.

9. Table 3: Please add the N.

**Reviewer**	**2**
**Name**	**Mackesy-Amiti, Mary Ellen**
**Affiliation**	**University of Illinois at Chicago College of Medicine**
**Date**	**11-Dec-2025**
**COI**	**None**

This paper reports findings on the prevalence of STI symptoms among PWID across 3 years of a national survey, and an analysis of correlates of STI symptom reporting among MWID in the most recent year of the survey. The subject matter is worthy of reporting however the paper is in need of significant improvement in clarity. In addition, I have concerns about the methods used to arrive at the final multivariable model. The discussion is generally well organized and there is appropriate discussion of limitations.

Introduction:

1. In the 3rd sentence, it is not clear if this refers to global or regional estimates.

2. The last sentence of 1st paragraph indicates a significant disparity between men and women in lifetime prevalence but it is not clear if this is a finding that has been observed repeatedly. This statement raises an issue that may be a distraction from the purpose of the paper.

3. Some revisions are suggested to improve flow and clarity (see attached markup).

Methods:

1. A large number of variables were identified as significant in bivariate analyses, and it is not clear how potential collinearity was handled in the backward regression variable selection process.

Results:

1. In general, reporting of results is not always clear. For example, it is not clearly stated when percentages refer to symptomatic PWID rather than the entire sample.

2. There are serious discrepancies in the data reported in Table 1, or it is not clear what is being reported.

Discussion:

1. Needs some discussion of why daily injection, needle sharing, and medications for opioid use disorder are included and what it means if they are significantly associated with STI symptoms while including sexual risk factors.

There are numerous minor typographical and grammatical errors throughout.

Table 4. Include chi-square in table.

**** The reviewer provided a marked copy with additional comments. Please contact the publisher for full details.****

VERSION 1 - AUTHOR RESPONSE

Reviewer: 1
Dr. Kyle Dack, University of Bristol
Comments to the Author:
Statistical review summary:
Overall this is a very well written paper. The methods are described clearly and the interpretation of the findings is balanced. However, the modelling building practice has a high risk of bias because it is data-driven without theoretical justification, and risks introducing confounding bias and inflated type 1 error. I recommend replacing this with an evidence-based approach to variable selection, such as discussed for example here: https://pmc.ncbi.nlm.nih.gov/articles/PMC6447501/ , https://pubmed.ncbi.nlm.nih.gov/37771042/

Response: We thank the reviewer for the thoughtful and constructive feedback, and for acknowledging the overall clarity and balance of our paper. We agree with the reviewer's caution regarding purely data-driven variable selection and the associated risks of bias. In our study, the selection of variables was guided by a structured, multi-step process specifically designed to balance theoretical justification with empirical screening—a common approach in exploratory epidemiological research.

First, candidate variables were identified through a targeted review of existing literature on associated factors of symptom-based sexually transmitted infections, ensuring our model was grounded in prior evidence (Kleinbaum & Klein, 2010; Sun et al., 2014). This list was then refined through consultation with our research team to ensure relevance and face validity. Finally, a conservative bivariable screening threshold ($p < 0.2$) was used to identify variables for inclusion in the multivariable model. This threshold is widely recommended to avoid prematurely excluding potentially important confounders or predictors in the initial stages of model building (2, 3).

Our goal was not to infer causation, but rather—as stated in our aims—to identify robust associations within a cross-sectional design and generate hypotheses for future longitudinal or interventional studies (Wang & Cheng, 2020). This approach is considered appropriate

and transparent when investigating associated factors in settings where prior evidence is evolving, and the focus is on thorough, rather than parsimonious, exploratory analysis.

To improve clarity, we have revised the Methods section to explicitly describe this three-step selection process (literature → expert consultation → statistical screening) and have added citations to methodological guidelines supporting this approach. We believe these revisions will help readers better understand the theoretical and practical rationale behind our variable selection.(4-6)

We added this text to the Methods section:
"A structured, hybrid approach to variable selection was used that integrated theoretical justification from the literature and team expertise with a pragmatic statistical criterion. This method is a recognized and conservative strategy for exploratory analysis of associated factors in cross-sectional studies, where the goal is to identify robust candidates for future causal investigation rather than to infer causation."

1. Study framing: The authors acknowledge in the discussion that the data type prevents causal interpretation because all measurements were taken at the same time. For the most part, the interpretation and reporting is aligned with this. However, the word "influence" is used in various parts of the manuscript which does imply causality. I suggest alternatives such as "correlate", "explain variance in", "linked to", etc.

Response: Thank you for your insightful comment. It has been addressed and corrected.

Abstract:
2. In the results where it states "Also in this year" it is not clear what year is being referred to.

Response: Thank you for this comment. It has been replaced with "in 2023".

Methods:
3. I suggest adding a "study overview" or "study design" section to begin the methods, giving brief details of the design (repeated cross-sectional?), primary outcome, study dates, and participants. This is described in more detail in the STROBE elaboration document, and helps readers quickly understand the basic elements of the study.

Response: Thank you for this suggestion. We have added a new subheading in the Methods section entitled "Study Overview," which reads as follows: "These repeated cross-sectional surveys were conducted as three national integrated bio-behavioral surveillance surveys (IBBSS) in 2010 (n=2,546), 2014 (n=2,399), and 2023 (n=2,379) across 10-14 major Iranian cities. Participants were PWID with a history of injection within the past 12 months and aged 18 years or older. They were recruited through facility-based (2010 and 2014) and respondent-

driven sampling (2023) methods across different years. The prevalence of symptom-based STIs was assessed, with self-reported STI symptoms in 2023 defined as the primary outcome."

4. Overall, the methods are clear, reasonable, and transparent. My main concern is the variable selection strategy. Section 2.4 lists numerous variables, but there is not a distinction between independent variables theorized to correlate with the outcome, and covariates included to minimize confounding bias.

The current approach screens all variables by bivariate significance, and includes the significant variables in the final multivariable model. This is essentially a hypothesis-free approach, it is data-driven and does not account for confounding bias. It also introduces the risk of other forms of bias such as overfitting and inflated type 1 error. Variable selection should instead by guided by prior evidence and theory, with covariates selected to minimize confounding bias. If prior evidence/theory is not available, I recommend explicitly reframing the analysis as exploratory and descriptive, due to the higher risk of bias.

Response: We thank the reviewer for this valuable comment. We agree with the reviewer's caution regarding purely data-driven variable selection and the associated risks of bias. As noted earlier in response to the 1st comment, the selection of variables was guided by a structured, multi-step process specifically designed to balance theoretical justification with empirical screening—a common approach in exploratory epidemiological research. Section 2.5 was revised and it reads as follows: "Conceptual Framework: Our variable selection was guided by the Risk Environment Framework, which conceptualizes drug-related harms, including STIs, as products of the interaction between individuals and their physical, social, economic, and policy environments (7-9). This framework is particularly relevant for understanding STI risk among PWID, as it accounts for both injection-related and sexual risk pathways and their shared structural determinants. Following this framework, we categorized variables into five domains. The physical environment domain reflects access to health services and harm reduction infrastructure, assessed through ever having been tested for HIV (yes or no), ever having been tested for HCV (yes or no), and whether participants received harm reduction services in the last 12 months (yes or no). The social environment domain encompasses social relationships, structural vulnerability, and marginalization. Marital status was categorized as single, married or living with a sexual partner or temporary marriage, or separated/widowed/divorced. Social marginalization was assessed through history of homelessness (yes or no) and incarceration (yes or no) in the last 12 months. Sexual network characteristics included having multiple sexual partnerships in the last 6 months (yes or no) and ever having experienced sex with men (yes or no). Human capital variables included age, dichotomized as less than 30 years or 30 years and older, and educational level, categorized as less than high school versus high school or academic education. The economic environment domain reflects financial capacity and economic vulnerability, assessed through monthly income dichotomized at the threshold of 70 USD (less than 70 USD or 70 USD and above). The

policy environment domain captures the reach of programmatic interventions. Receipt of harm reduction services in the last 12 months served as a proxy for national harm reduction policy implementation, while ever having been tested for HIV and HCV reflected the extent to which national testing and screening guidelines have reached PWID. Several variables in this domain naturally overlap with the physical environment, as policy decisions determine service availability while physical infrastructure mediates policy delivery. The proximal risk behaviors domain encompasses individual behaviors shaped by the broader risk environment that directly affect STI transmission. Sexual risk behaviors included having multiple sexual partnerships in the last 6 months (yes or no), last unprotected sex with casual partners in the last 6 months (yes or no), and ever having experienced sex with men (yes or no). Injection risk behaviors comprised type of main injected drug in the last 6 months (opioids or stimulants), daily injection in the last 6 months (yes or no), and history of sharing needles, syringes, or equipment in the last 6 months (yes or no). While sexual behaviors represent direct STI transmission pathways, injection-related variables were retained based on prior evidence that injection practices and sexual risk behaviors are interconnected through behavioral syndemics effects, shared structural determinants, and potential biological interactions environments (7-9)."

5. "A multivariable logistic regression model was performed to report STI symptoms and adjusted odds ratios (AORs)." I suggest specifying the full model parameterization – i.e. which variables were included.

Response: Thank you for your suggestion. In the "Data Analysis" section, bivariable screening identified the following variables with $p<0.2$ for inclusion in multivariable modeling: marital status, educational level, monthly income, history of homelessness in the last 12 months, multiple sexual partnership in the last 6 months, last unprotected sex with casual partners in the last 6 months, ever experienced sex with men, ever HIV testing, ever HCV testing, type of main injected drug in the last 6 months, daily injection in the last 6 months, sharing needles, syringes, and equipment in the last 6 months. These were entered simultaneously into a backward stepwise logistic regression model. Variables were removed sequentially if their removal did not significantly worsen model fit (likelihood ratio test $p>0.05$). The process continued until all remaining variables had $p<0.05$ or their removal significantly degraded model fit. We did not include interaction terms in the final model.

6. It is not clear how time is handled in the analyses. It looks to me like the chi-square bivariate comparisons, and the regression models were all limited to 2023 data. I recommend stating this for clarity, and to reassure readers because my initial concern was that the models may pool data from the 3 surveys, which would break the assumption of non-clustered data.
I suggest also clarifying what the 2010 and 2013 data was used for, i.e. the trend analyses shown in section 3.1.

Response: Thank you very much for this comment. The 2010,2014 and 2023 data was used for trend analysis shown in section 3.2. We only used 2023 data for comparison and logistic regression models. To clarify this point, we added the following sentence to the Methods section under the "Data Analysis" subheading: "The 2010,2014 and 2023 data was used for trend analysis and for determining predictors of symptom-based STIs, we used data from BBSS 2023."

Results

7. Please begin by repeating the sample sizes, and the basic characteristics of the cohorts, to align with STROBE requirements.

Response: Thank you very much for your guidance. For clarification, we added overall information based on Table 1 at the beginning of the Results section, which reads as follows: "In the present study, 7,325 PWID were recruited across three survey rounds: 2,546 in 2010, 2,399 in 2014, and 2,379 in 2023. The majority were men (7,094, 96.8%) The mean age was 37.9 years (SD 9.6), and 5,524, 77.9% had high school education and less. Among sexually active men (defined as any sexual contact in the last 6 months), the sample sizes were 1,050 in 2010, 1,045 in 2014, and 1,191 in 2023."

8. Section 3.5. I suggest adding more information in this section. Currently it does not add much which cannot already be found in the corresponding table, and again I do not think it fully adheres to STROBE in relation to comprehensive explanation of the statistics. E.g. "Had multiple sexual partnership in the last 6 months (AOR: 4.42; 95% CI: 1.31 – 14.88);" – explain the comparison category, explain the meaning of a 4.42 odds ratio, acknowledge the very wide confidence interval which suggests uncertainty in the precise point estimate, but indicates evidence of some form of increased odds. There is also no comment on the variables which were not significant, which risks introducing reporting bias.

Response: Thank you very much for this valuable comment. We agree that Section 3.5 required further elaboration to fully adhere to STROBE recommendations. We have revised the section to provide a more comprehensive explanation of the statistical findings. Specifically, we now clarify the comparison categories for each variable, interpret the meaning of the adjusted odds ratios, and acknowledge the wide confidence intervals, which suggest uncertainty in the precise point estimates but indicate evidence of increased odds. In addition, we have added comments on variables that were not statistically significant to avoid potential reporting bias. It reads as follows:

"According to multivariable logistic regression model, the following variables increased the odds of symptom-based STIs among MWID: being separated, widow or divorce (AOR: 2.56;

95% CI: 1.14 – 5.76) versus being single; had multiple sexual partnership in the last 6 months (AOR: 4.08; 95% CI: 1.22 – 13.65) versus had single partner; had last unprotected sex with casual partners in the last 6 months (AOR: 2.41; 95% CI: 1.27 – 4.58) versus last protected sex with casual partners; had ever sex with men (AOR: 2.98; 95% CI: 1.36 – 6.54) versus never sex with men could predict the symptom-based STI among MWID. Also, stimulants as main injected drug in the last 6 months (AOR: 2.579; 95% CI: 1.08–6.14) versus opioid injection; and sharing needles, syringes, and equipment in the last 6 months (AOR: 2.84; 95% CI: 1.53 – 5.27) versus keeping them determined as predictors for symptom-based STI in this population (**Table 3**). Although the odds ratio was elevated, the wide confidence interval indicates considerable uncertainty around the precise estimate. In this level, following variables were not significant: educational level, monthly income, history of homelessness in the last 12 months, ever HIV testing, ever HCV testing, and daily injection drug in the last 6 months."

9. Table 3: Please add the N.

Response: Thank you for your suggestion. It was done.

Reviewer: 2

Dr. Mary Ellen Mackesy-Amiti, University of Illinois at Chicago College of Medicine
Comments to the Author:

This paper reports findings on the prevalence of STI symptoms among PWID across 3 years of a national survey, and an analysis of correlates of STI symptom reporting among MWID in the most recent year of the survey. The subject matter is worthy of reporting however the paper is in need of significant improvement in clarity. In addition, I have concerns about the methods used to arrive at the final multivariable model. The discussion is generally well organized and there is appropriate discussion of limitations.

Response: Thanks for your thoughtful review and constructive feedback. In response to your concerns regarding clarity, we have carefully revised the manuscript to improve readability and precision throughout, particularly in the Methods and Results sections. Regarding the multivariable model, we have clarified our variable selection process and added details on how collinearity was assessed and addressed. Please see our detailed responses below.

Introduction:

1. In the 3rd sentence, it is not clear if this refers to global or regional estimates.

Response: Thank you for this comment. As it was related to global statistics, we have revised the text to provide clarification and it read as follow: "Based on global information, in 2020, the daily incidence of new STI cases exceeded one million among adults aged 15-49 years".

2. The last sentence of 1st paragraph indicates a significant disparity between men and women in lifetime prevalence but it is not clear if this is a finding that has been observed repeatedly. This statement raises an issue that may be a distraction from the purpose of the paper.

Response: Thank you for your insightful comment. Yes, this sentence was based on the findings of an article. By highlighting this difference, we have laid the groundwork for the main purpose of the study In Iran, the lifetime prevalence of STI symptoms is substantially higher among women (>80%) than men (33%) (10), likely reflecting both biological susceptibility and gender differences in healthcare-seeking behaviors. However, among PWID—who are predominantly male (95% in Iran)—STI epidemiology remains understudied.

3. Some revisions are suggested to improve flow and clarity (see attached markup).
Response: Thank you very much. We have applied these changes in the main text.

Typos in the Abstract section were corrected.

Background section:

globally?

Response: Yes, the statistic is based on global reports.

Curious why the disparity? Has this disparity been observed before? May want to note reasons such as reporting bias? or might want to take this out since the focus is on men this is distracting? Response: The prevalence of STIs among women is generally higher than among men because of biological susceptibility (female anatomy makes transmission easier), asymptomatic infections that delay diagnosis, and social/structural factors such as stigma and unequal access to care. As previously stated, this sentence was based on the findings of an article. By highlighting this difference, we have laid the groundwork for the main purpose of the study. the lifetime prevalence of STI symptoms is substantially higher among women (>80%) than men (33%) (10), likely reflecting both biological susceptibility and gender differences in healthcare-seeking behaviors. However, among PWID—who are predominantly male (95% in Iran)—STI epidemiology remains understudied.

Missing word?

Response: The corrected phrase is "understudies' issue despite".

Needs rephrasing, make it sound like contaminated injection equipment can cause STIs?

Response: We appreciate the reviewer's comment. We have revised the sentence to clarify that contaminated injection equipment is a source of blood-borne infections, while risky sexual practices are the source of sexually transmitted infections. The revised text now reads: "PWID face dual risk pathways: blood-borne infections through contaminated injection

equipment, and sexually transmitted infections through unsafe sexual practices, making them a critical population for surveillance and intervention.”

Out of place, flows better without this sentence.

Response: Thank you, we agree with deleting this sentence.

Method section:

Out of order:

Response: Thank you for this comment. Inclusion criteria for last round are mentioned separately and they read as follows: “Inclusion criteria were defined as those aged 18 years or older, had a history of injection in the past 12 months, resided in the city of the study, expressed their consent for participating in the study, and possessing a valid coupon (except for seeds).”

Ad ed: The spelling error has been corrected. It reads “Well-networked participants”.

Result section:

Higher in 2010 compared to which year? Which year was 66.4%?

Response: We revised this section. The prevalence of symptoms of STIs among symptomatic PWID was reported. The prevalence of symptoms of STIs was revised because previous reports were based on PWID who reported symptoms of STIs in the last 12 months, but some of them were not sexually active in the last 6 months. Therefore, we changed the denominator, and it was restricted to PWID with a history of sexual activities in the last 6 months. It read as follows: “Over time, the number of PWID who had a history of sexual contact in the last 6 months was 1,124 (44.1%) in 2010, 1,124 (46.8%) in 2014, and 1,257 (52.8%) in 2023. During these years, the prevalence of symptom-based STI was approximately stable [2010: 10.1% (95% CI: 8.35 – 11.96); 2014: 10.5% (95% CI: 8.76 –12.43) and 2023: 10.3% (95% CI: 8.63 – 12.07)]. The prevalence of genital ulcers and excessive discharge among symptomatic PWID was 64.6% and 76.1%, respectively, in 2010. While in 2014, genital ulcers were still prevalent among symptomatic PWID (94.9%), but pain and burning were common symptoms among symptomatic PWID in 2023 (90.7%) and genital ulcers and genital warts had lower prevalence among them in this year (11.6% and 0.8%, respectively)”.

Also, should note these are percentages among symptomatic PWID.

Response: The comment has been addressed.

Assume this should be among symptomatic PWID.

Response: That’s right. The comment has been addressed.

Symptomatic PWID

Response: The comment has been addressed.

Grammar errors in result section were corrected, and some parts of this section were revised.

Discussion section:

Grammar errors in the discussion section were corrected.

Were MSM less likely to seek care at medical clinics?

Response: Thank you for this comment. This paragraph discussed seeking care behaviors among PWID generally. We did not assess seeking care behaviors based on sexual practices.

Table section:

Grammar errors in the table were corrected. Analysis errors were checked and corrected. In the present study, the symptoms of STIs were checked in 12 months, but the sexual activities were checked in the last 6 months. We changed the denominator, and for all years, only PWID who had sexual activities in the last 6 months were considered. Among symptoms of STIs, "pain and burning" and "genital warts" were not asked of participants in 2010 and 2014. This matter was mentioned in the limitation section, and in the table, we reported "NR". We also added the chi-squared value to Table 1.

Methods:

1. A large number of variables were identified as significant in bivariate analyses, and it is not clear how potential collinearity was handled in the backward regression variable selection process.

Response: We appreciate this comment. The data analysis section was revised and it reads as follows: "The analytical population in this study included PWID who reported sexual contact in the last 6 months with any partners. Guided by the Risk Environment Framework described above, we employed a structured, hybrid approach to variable selection that integrated theoretical justification with empirical screening (7, 8). The framework provided the conceptual foundation for organizing variables across five domains (physical, social, economic, and policy environments, plus proximal risk behaviors), ensuring that our variable selection was grounded in established theory about how environmental contexts shape health risks among PWID. Within this framework, candidate variables representing each environmental domain were identified through targeted review of existing literature on STI risk factors among PWID, with particular attention to systematic reviews and WHO guidance on surveillance among key populations (11). This literature-informed list was then refined through consultation with our multidisciplinary research team, including epidemiologists, infectious disease specialists, and harm reduction practitioners, to ensure contextual relevance to Iranian PWID populations, data availability across survey rounds, and measurement validity in face-to-face interviews.

Data were described using mean and standard deviation for continuous variables and frequency with 95% confidence intervals for categorical variables. Missing values were not inferred. The 2010,2014 and 2023 data was used for trend analysis and for determining

predictors of symptom-based STIs, we used data from BBSS 2023. Following the theoretical grounding described above, we applied a conservative bivariable screening threshold of p<0.20 through bivariable logistic regression to compare variables in terms of STI symptom reporting status. This statistical criterion, widely recommended in epidemiological practice (12), avoids prematurely excluding potentially important confounders or predictors in exploratory analysis while maintaining a degree of empirical parsimony. Variables meeting this threshold were entered into a backward stepwise logistic regression model (13), with the likelihood ratio test used to compare the reduced model with the full model at each step. Prior to model finalization, we assessed collinearity in the full multivariable model containing all variables with p<0.20 from bivariable screening using variance inflation factors (VIFs) and tolerance statistics. We used a VIF threshold of 10, which is recommended for epidemiological regression models (14). All VIF values ranged from [1.04-1.25], with a median VIF of 1.12, indicating no substantial statistical collinearity. In addition to statistical assessment, we evaluated conceptual overlap between variables. Several variable clusters measured related constructs (e.g., sexual risk behaviors, healthcare engagement, harm reduction service utilization). We retained conceptually related variables when they captured distinct dimensions of the risk environment (e.g., daily injection frequency vs. equipment sharing as separate measures of injection risk). The multivariable logistic regression model was used to estimate adjusted odds ratios for reporting STI symptoms.

This three-step process (theoretical framework followed by literature review and expert consultation, then statistical screening) balances the need for theoretical justification with the pragmatic realities of exploratory research in contexts where the evidence base is still evolving. Importantly, our goal was not to infer causation, but rather, as stated in our aims, to identify robust associations within a cross-sectional design and generate hypotheses for future longitudinal or interventional studies. This method represents a recognized and conservative strategy for exploratory analysis of associated factors in cross-sectional surveillance studies, where comprehensive exploration of risk factors is prioritized to inform public health programming. To examine the association between HIV, HCV, and HBV status and symptoms of STIs, we performed chi-square tests. Statistical significance was set at p-value of 0.05 or less. All analyses were conducted using Stata version 17."

Results:

1. In general, reporting of results is not always clear. For example, it is not clearly stated when percentages refer to symptomatic PWID rather than the entire sample.

Response: Thank you for your valuable comment. In the Results section, in the second paragraph of "Overall trends in symptom-based STI prevalence," we replaced "PWID" with "symptomatic PWID." In the other parts of the manuscript, we considered all MWID.

2. There are serious discrepancies in the data reported in Table 1, or it is not clear what is being reported.

Response: It was an insightful comment, thank you. In this table, we present the total number of PWID, the number with a recent history of sexual contact, the number reporting recent STI symptoms, and the prevalence of STI among sexually active PWID. Subsequently, the frequency of specific STI symptoms—including pain and burning, genital ulcers, excessive discharge, and genital warts—is reported. We also provide information on treatment-seeking behavior and, among those who sought treatment, the type of treatment received. All data are drawn from three survey rounds and are reported both in total and separately for men.

Discussion:

1. Needs some discussion of why daily injection, needle sharing, and medications for opioid use disorder are included and what it means if they are significantly associated with STI symptoms while including sexual risk factors.

Response: Thank you for your guidance. PWID who engage in daily injection and condomless sex face a dual pathway of risk for sexually transmitted infections. Daily injection increases exposure to blood-borne pathogens through needle sharing, while condomless sex directly facilitates STI transmission. Together, these overlapping behaviors act synergistically, contributing to the elevated burden of STIs in this population. We added a paragraph to the Discussion section. It reads as follows:

"According to our findings, MWID with daily injection behaviors in the past six months had higher odds of STIs. This result is consistent with previous studies and confirms the influence of substance use behaviors on sexual risk behaviors. Prior research has also shown that daily injection drug use is linked to a higher likelihood of reporting an STI (15). Furthermore, daily injection could be associated with increased needle sharing (15), which elevates the risk of blood-borne infections and STIs. Condomless commercial sex is also more common among PWID who inject daily (16), further compounding their vulnerability. In addition, PWID who share needles and syringes are less likely to use condoms consistently with different types of female partners (17), suggesting that overlapping high-risk injection and sexual practices contribute synergistically to the elevated burden of STIs in this population. To mitigate the elevated burden of STIs among PWID with daily injection and condomless sex, integrated harm-reduction and sexual health interventions—including needle exchange, opioid substitution therapy, condom distribution, and routine STI screening—are recommended, alongside structural measures to reduce stigma and improve service accessibility."

There are numerous minor typographical and grammatical errors throughout.

Response: Thank you for pointing this out. We have carefully reviewed the entire manuscript and corrected typographical and grammatical errors throughout. In addition, we have refined sentence structures and improved clarity to ensure the text reads more smoothly. We believe these revisions enhance the overall quality and readability of the manuscript.

Table 4. Include chi-square in table.

Response: Thank you for this comment. We inserted another column into Table 4 and added the Chi-squared values.

References

1. Khezri M, Tavakoli F, Mehmandoost S, Sharafi H, Zamani O, Bazrafshani MS, et al. Engagement in hepatitis C virus cascade of care and factors associated with testing among people who inject drugs in Iran. Harm Reduction Journal. 2025;22(1):1-10.
2. Bursac Z, Gauss CH, Williams DK, Hosmer DW. Purposeful selection of variables in logistic regression. Source code for biology and medicine. 2008;3(1):17.
3. Mickey RM, Greenland S. The impact of confounder selection criteria on effect estimation. American journal of epidemiology. 1989;129(1):125-37.
4. Kleinbaum DG, Klein M. Logistic regression: a self-learning text: Springer; 2002.
5. Sun G-W, Shook TL, Kay GL. Inappropriate use of bivariable analysis to screen risk factors for use in multivariable analysis. Journal of clinical epidemiology. 1996;49(8):907-16.
6. Wang X, Cheng Z. Cross-sectional studies: strengths, weaknesses, and recommendations. Chest. 2020;158(1):S65-S71.
7. Rhodes T. The ‘risk environment’: a framework for understanding and reducing drug-related harm. International journal of drug policy. 2002;13(2):85-94.
8. Rhodes T. Risk environments and drug harms: a social science for harm reduction approach. Elsevier; 2009. p. 193-201.
9. Strathdee SA, West BS, Reed E, Moazen B, Azim T, Dolan K. Substance Use and HIV Among Female Sex Workers and Female Prisoners: Risk Environments and Implications for Prevention, Treatment, and Policies. J Acquir Immune Defic Syndr. 2015;69 Suppl 2(0 1):S110-7.
10. Dashtaki NM, Askari F, Fararouei M, Ghaem H, Dianati-Nasab M. Sexual Practices and Their Associates in Iranian Female Sex Workers. Sexuality Research and Social Policy. 2025;22(3):1249-55.
11. Organization WH. Consolidated guidelines on HIV, viral hepatitis and STI prevention, diagnosis, treatment and care for key populations: World Health Organization; 2022.
12. Katz MH. Multivariable Analysis: A Practical Guide for Clinicians. 2 ed. Cambridge: Cambridge University Press; 2006.
13. Bursac Z, Gauss CH, Williams DK, Hosmer DW. Purposeful selection of variables in logistic regression. Source Code Biol Med. 2008;3:17.
14. Vatcheva KP, Lee M, McCormick JB, Rahbar MH. Multicollinearity in regression analyses conducted in epidemiologic studies. Epidemiology (Sunnyvale, Calif). 2016;6(2):227.
15. Rusch MLA. Substance use and sexually transmitted infections, barriers to sexual health care and the role of enhanced outreach services on STI trends among an inner-city population in Vancouver, Canada: University of British Columbia; 2006.
16. Su S, Zhang L, Cheng F, Li S, Li S, Jing J, et al. Association between recreational drug use and sexual practices among people who inject drugs in Southwest China: a cross-sectional study. BMJ open. 2018;8(6):e019730.
17. Sharma SK. P4. 95 Declining trends in risk behaviours and hiv/stis among injecting drug users in india: findings from large scale bio-behavioural surveys from three high prevalent states. BMJ Publishing Group Ltd; 2017.

VERSION 2 - REVIEW

**Reviewer**	**1**
**Name**	**Dack, Kyle**
**Affiliation**	**University of Bristol, MRC Integrative Epidemiology Unit**
**Date**	**30-Mar-2026**
**COI**	

Thank you to the authors for the detailed responses, and the clarifications made to the manuscript.

I remain concerned that the modelling strategy does not follow recommended practices for minimizing bias in estimates - it uses univariate statistical screening, and backward stepwise regression.

While the authors added clarification, and describe it as a hybrid approach integrating both theory and empirical screening, ultimately, this does not seem accurate: in practice the statistical significance dominates - regardless of the strength of evidence or theory, variables were dropped if they did not meet the significance threshold.

There is a significant body of literature recommending against stepwise and significance-based screening, due to the conflict between these approaches and evidence-based controlling of confounding bias, potential overfitting, and the interpretability of the final multivariable model. E.g., https://pmc.ncbi.nlm.nih.gov/articles/PMC10952199/, https://onlinelibrary.wiley.com/doi/10.1016/j.pmrj.2013.07.007, https://academic.oup.com/ije/article/50/2/620/6012812

If the aim is to generate hypotheses, and this is an exploratory analysis where the relevant variables are unclear, this approach may be justified. However, this framing should be applied throughout the manuscript. In particular, (1) indicating in the abstract that the study design is exploratory, (2) explaining the rationale for an exploratory rather than fully evidence-based approach in the introduction (e.g. is there a lack of evidence for which to determine the confounders?), and (3) adopting more cautious interpretation in the discussion, by more directly discussing as a limitation that the modelling approach may not minimizing all potential biases (confounders could be dropped, potential cumulative impact of dropped variables, risk of mediators)

Overall the manuscript is very well written, and I encourage the authors to revise and ensure all sections of the manuscript align regarding the above.

**Reviewer**	**2**
**Name**	**Mackesy-Amiti, Mary Ellen**
**Affiliation**	**University of Illinois at Chicago College of Medicine**
**Date**	**14-Apr-2026**
**COI**	

The manuscript is substantially improved; however, it still needs careful proofreading.

A response to my comment about addressing the meaning of associations between injection risk behaviors and STI symptoms in the discussion was included in the response letter but was not in the revised manuscript. The statement that "daily injection could be associated with increased needle sharing (14), which elevates the risk of bloodborne infections and STIs" should be omitted, as needle sharing in itself does not increase the risk of STIs.

More to the point, the paragraph does not clearly address the issue I was raising, which is that sharing syringes and other injection equipment remained significant in the multivariable model that included sexual risk behaviors. This suggests that there are additional sexual risk behaviors associated with syringe and equipment sharing that are not accounted for in the model.

VERSION 2 - AUTHOR RESPONSE

Reviewer: 1

Dr. Kyle Dack, University of Bristol

Comments to the Author:

Thank you to the authors for the detailed responses, and the clarifications made to the manuscript.
I remain concerned that the modelling strategy does not follow recommended practices for minimizing bias in estimates - it uses univariate statistical screening, and backward stepwise regression.
While the authors added clarification, and describe it as a hybrid approach integrating both theory and empirical screening, ultimately, this does not seem accurate: in practice the statistical significance dominates - regardless of the strength of evidence or theory, variables were dropped if they did not meet the significance threshold.
There is a significant body of literature recommending against stepwise and significance-based screening, due to the conflict between these approaches and evidence-based controlling of confounding bias, potential overfitting, and the interpretability of the final multivariable model.
E.g., https://pmc.ncbi.nlm.nih.gov/articles/PMC10952199/, https://onlinelibrary.wiley.com/d

oi/10.1016/j.pmrj.2013.07.007, https://academic.oup.com/ije/article/50/2/620/6012812
If the aim is to generate hypotheses, and this is an exploratory analysis where the relevant variables are unclear, this approach may be justified. However, this framing should be applied throughout the manuscript. In particular, (1) indicating in the abstract that the study design is exploratory, (2) explaining the rationale for an exploratory rather than fully evidence-based approach in the introduction (e.g. is there a lack of evidence for which to determine the confounders?), and (3) adopting more cautious interpretation in the discussion, by more directly discussing as a limitation that the modelling approach may not minimizing all potential biases (confounders could be dropped, potential cumulative impact of dropped variables, risk of mediators)
Overall the manuscript is very well written, and I encourage the authors to revise and ensure all sections of the manuscript align regarding the above.
Response: We thank the reviewer for this thoughtful and constructive comment. We agree that our previous description of the modelling strategy as a "hybrid" approach may have overstated its role in confounder control, since the final model was ultimately influenced by statistical selection criteria. We also agree that bivariable screening and backward stepwise regression are not recommended approaches for minimizing confounding bias or for producing causal estimates. We have therefore revised the manuscript throughout to frame the analysis explicitly as exploratory and hypothesis-generating, rather than as a fully evidence-based confounder-control strategy.

To address the reviewer's specific concerns, we made the following revisions:

Abstract: We now describe the study as an exploratory analysis and revised the objective to state that we aimed to "explore factors associated with" STI symptom reporting, rather than identify predictors. We also added cautionary language indicating that the findings should be interpreted as hypothesis-generating rather than causal.

Introduction: We added a clearer rationale for the exploratory approach. Specifically, we now explain that evidence on symptom-based STI reporting among MWID in Iran is limited and heterogeneous, and that the available literature is insufficient to prespecify a complete causal framework or minimally sufficient adjustment set for this outcome and population.

Methods: We removed language describing the modelling approach as "hybrid," "conservative," or "recognized." The statistical analysis section now explicitly states that bivariable screening and backward stepwise regression were used to develop a parsimonious exploratory model of adjusted associations, not to estimate causal effects or identify a minimally sufficient adjustment set.

Results: We revised the Results to avoid causal or predictive wording. Terms such as "predictors," "increased the odds," "could predict," and "determined as predictors" were replaced with "associated with higher odds" and "retained in the final exploratory model."

The title of the multivariable results section and Table 3 were also revised to emphasize the exploratory nature of the analysis.

Discussion and Limitations: We adopted a more cautious interpretation throughout. We now explicitly state that the findings should not be interpreted as causal effects or definitive predictors. The limitations section was expanded to acknowledge that the modelling approach may have excluded potential confounders that did not meet statistical thresholds, that cumulative exclusion of variables may have contributed to residual confounding, and that some retained variables may be mediators or markers of broader unmeasured risk environments rather than independent causal determinants.

Conclusion: We revised the conclusion to clarify that the associated factors identified in this study should be used to inform future research and intervention planning, rather than interpreted as definitive causal determinants of STI risk.

Overall, we have aligned the Abstract, Introduction, Methods, Results, Discussion, Limitations, and Conclusion with an exploratory, hypothesis-generating interpretation of the analysis. We appreciate the reviewer's guidance, which improved the methodological transparency and interpretability of the manuscript.

Reviewer: 2
Dr. Mary Ellen Mackesy-Amiti, University of Illinois at Chicago College of Medicine
Comments to the Author:
The manuscript is substantially improved; however, it still needs careful proofreading.

A response to my comment about addressing the meaning of associations between injection risk behaviors and STI symptoms in the discussion was included in the response letter but was not in the revised manuscript. The statement that "daily injection could be associated with increased needle sharing (14), which elevates the risk of bloodborne infections and STIs" should be omitted, as needle sharing in itself does not increase the risk of STIs.

More to the point, the paragraph does not clearly address the issue I was raising, which is that sharing syringes and other injection equipment remained significant in the multivariable model that included sexual risk behaviors. This suggests that there is additional sexual risk behaviors associated with syringe and equipment sharing that are not accounted for in the model.

Response: We thank the reviewer for these helpful comments. We have carefully proofread the manuscript and revised wording throughout to improve clarity, grammar, consistency, and readability.

We also agree that our previous wording could be interpreted as implying that needle or equipment sharing directly increases STI transmission risk. We have therefore revised the

Discussion to address the reviewer's key point more directly: sharing needles, syringes, or other injection equipment remained associated with STI symptom reporting even after adjustment for measured sexual risk behaviours. We now clarify that this association should not be interpreted as evidence of direct STI transmission through injection equipment. Instead, it may reflect unmeasured sexual risk behaviours, overlapping sexual and injection networks, unstable access to harm reduction services, or broader risk environments associated with both syringe/equipment sharing and STI symptoms.

It now reads: "**Injection-related characteristics were also retained in the final exploratory model. In particular, sharing needles, syringes, or other injection equipment was associated with STI symptom reporting even after adjustment for measured sexual behaviours included in the model. This association should not be interpreted as evidence that sharing injection equipment directly transmits STIs, such as gonorrhoea, chlamydia, or syphilis, which are primarily transmitted through sexual contact. Rather, equipment sharing may be a marker of broader risk environments, including higher-risk social or drug-use networks, unstable access to harm reduction resources, overlapping sexual and injection partnerships, or unmeasured sexual behaviours not captured in the survey. This interpretation is consistent with evidence that unsafe injection and sexual risk behaviours can co-occur among PWID. These findings suggest that STI prevention for MWID should not focus only on individual sexual behaviors, but should also address the broader risk environment through integrated harm reduction, condom provision, STI testing or syndromic management, counselling, and linkage to care.**"
